# Time course of blood pressure control prior to lacunar TIA and stroke: Population-based study

**DOI:** 10.1212/WNL.0000000000005526

**Published:** 2018-05-15

**Authors:** Linxin Li, Sarah J.V. Welch, Sergei A. Gutnikov, Ziyah Mehta, Peter M. Rothwell, on behalf of the Oxford Vascular Study

**Affiliations:** From the Centre for Prevention of Stroke and Dementia, Nuffield Department of Clinical Neuroscience University of Oxford, UK.

## Abstract

**Objective:**

To determine the age-specific temporal trends in blood pressure (BP) before acute lacunar vs nonlacunar TIA and stroke.

**Methods:**

In a population-based study of TIA/ischemic stroke (Oxford Vascular Study), we studied 15-year premorbid BP readings from primary care records in patients with lacunar vs nonlacunar events (Trial of Org 10172 in Acute Stroke Treatment [TOAST]) stratified by age (<65, ≥65 years).

**Results:**

Of 2,085 patients (1,250 with stroke, 835 with TIA), 309 had lacunar events. In 493 patients <65 years of age, the prevalence of diagnosed hypertension did not differ between lacunar and nonlacunar events (46 [48.4%] vs 164 [41.2%], *p* = 0.20), but mean/SD premorbid BP (44,496 BP readings) was higher in patients with lacunar events (15-year records: systolic BP [SBP] 138.5/17.7 vs 133.3/15.0 mm Hg, *p* = 0.004; diastolic BP [DBP] 84.1/9.6 vs 80.9/8.4 mm Hg, *p* = 0.001), mainly because of higher mean BP during the 5 years before the event (SBP 142.6/18.8 vs 134.6/16.6 mm Hg, *p* = 0.0001; DBP 85.2/9.7 vs 80.6/9.0 mm Hg, *p* < 0.0001), with a rising trend (*p*_trend_ = 0.006) toward higher BP leading up to the event (<30-day pre-event SBP: 152.7/16.1 vs 135.3/23.1 mm Hg, *p* = 0.009; DBP 87.9/9.4 vs 80.8/12.8 mm Hg, *p* = 0.05; mean BP ≤1 year before the event 145.8/22.0 vs 134.7/16.1 mm Hg, *p* = 0.001; 86.1/10.7 vs 80.4/9.8 mm Hg, *p* = 0.0001). Maximum BP in the 5 years before the event was also higher in patients with lacunar events (SBP 173.7/26.6 vs 158.6/23.2 mm Hg, *p* = 0.0001; DBP 102.3/12.9 vs 94.2/11.2 mm Hg, *p* < 0.0001), as was persistently elevated BP (≥50% SBP >160 mm Hg, odd ratio 4.95, 95% confidence interval 1.99–12.31, *p* = 0.0002). However, no similar differences in BP were observed in patients ≥65 years of age.

**Conclusion:**

Recent premorbid BP control is strongly temporarily related to acute lacunar events at younger ages, suggesting a direct role of BP in accelerating causal pathology and highlighting the need to control hypertension quickly.

Usual blood pressure (BP) is a well-established risk factor for stroke.^1–4^ However, there is conflicting evidence of the direct role of BP in the etiology of lacunar stroke. While some studies found that previous hypertension was most common in patients with lacunar stroke^5–7^ and that it is predicts recurrent stroke most strongly in lacunar patients,^8^ others suggested an equal prevalence of hypertension and population-attributable fraction in lacunar vs nonlacunar stroke.^9,10^ However, most previous studies were hospital based; the majority used only history of diagnosed hypertension or a single BP often taken some years before the event or after the event^5–10^; many included old or silent lacune on brain imaging^11–13^; and none studied the temporal change of BP leading up to the event.

In a previous study, we showed that deep intracerebral hemorrhage was more closely associated with a recent increase of premorbid BP than lobar intracerebral hemorrhage, and we previously hypothesized that the same might also be true for lacunar vs nonlacunar ischemic stroke.^14^ It is likely, however, that any such temporal trends in premorbid BP in lacunar events will be most marked at younger ages, as has been shown for the overall association between BP and small vessel disease^15,16^ and as is suggested by the strong association with hypertension in the early autopsy studies done predominantly in young patients with lacunar stroke,^17^ whereas a higher rate of chronic arteriosclerotic or atheromatous pathology would be expected at older ages.^18^

In the absence of similar previous studies, we therefore aimed to determine the age-specific temporal trends in BP before acute lacunar vs nonlacunar TIA and ischemic stroke using 15-year premorbid BP measurements from primary care records in a population-based cohort.

## Methods

The Oxford Vascular Study (OXVASC) is an ongoing population-based study of the incidence and outcome of all acute vascular events.^19^ The study population comprises all 92,728 individuals, regardless of age, registered with ≈100 general practitioners in 9 general practices in Oxfordshire, UK.^20^ The multiple overlapping methods used to achieve near-complete ascertainment of all individuals with TIA or ischemic stroke are detailed in the supplemental data (e-methods, links.lww.com/WNL/A447) and have been reported previously.^19,20^ This analysis includes consecutive cases with a first TIA or ischemic stroke from April 1, 2002, to March 31, 2014.

Demographic data; vascular risk factors (previous hypertension, previous diabetes mellitus, previous atrial fibrillation, history of smoking, previous hyperlipidemia); history of cerebrovascular, coronary, or peripheral vascular disease; family history of stroke; and premorbid use of preventive treatment (antiplatelet agent, anticoagulation, lipid-lowering drug, and antihypertensive agent) were collected from face-to-face interview and cross-referenced with primary care records.^20^ Detailed clinical history was recorded in all patients, including the first postevent BP. Patients routinely had CT or MRI brain imaging, intracranial and extracranial vascular imaging, 12-lead ECG, and standard blood tests. Transthoracic echocardiography and long-term cardiac monitoring (e.g., 24-hour or 5-day ambulatory ECG) were done when clinically indicated.^20^ All cases were reviewed by a senior neurologist (P.M.R.), and stroke etiology was classified according to the modified Trial of Org 10172 in Acute Stroke Treatment (TOAST) criteria.^20,21^ Acute lacunar events were classified only in patients with no evidence of large artery, cardioembolic, or other rare etiology but fulfilled either imaging evidence of a single and clinically relevant acute subcortical infarction <20 mm, including acute lacunar lesion within the territory of brainstem penetrating arteries or clinical lacunar syndrome with no cerebral cortical dysfunction and normal CT/MRI. Given the potential for bias, risk factors such as hypertension and diabetes mellitus were not included in the criteria of lacunar events.^9^

Study nurses reviewed lifelong patient records held in primary care and extracted all premorbid BP readings with dates up to 15 years before the event in a standardized manner. We extracted data from both paper and computer records. Most readings were taken in the doctor's surgery by the physician or the practice nurse for screening purposes, regular review, or an episode of minor illness. Measurements made during previous hospital admissions, often for major illness, were not recorded. We also excluded measurements made in primary care at the time of any previous TIA or stroke.^14^

### Statistical analyses

Values are reported as absolute numbers with percentages for categorical variables and as means with SDs for continuous variables.

Premorbid BP measurements were presented as long-term average BP (mean BP taking into account all measurements before defined time points in patients with at least 1 premorbid BP measurement) and long-term BP variability (calculated in patients who had ≥4 premorbid BP measurements and presented as maximum BP, coefficient of variation [CV; 100 × SD/mean], and percentages of BP ≥50% above the target [systolic BP (SBP) 140/160/180 mm Hg; diastolic BP (DBP) 90/100/110 mm Hg]).

All analyses were stratified by age <65 and ≥65 years. We first compared the frequency of previous hypertension and premorbid use of antihypertensive agents in patients with lacunar vs nonlacunar events using the χ^2^ test. We then compared the acute postevent BPs and the long-term average BP (15-year mean BP, mean BP ≤5 years before the event, mean BP >5 years before the event) in patients with lacunar vs nonlacunar events using the *t* test. Trend of long-term BP change over time (i.e., >10, 5–10, 1–5, and ≤1 year and ≤30 days) in patients with lacunar vs nonlacunar events was assessed with the mixed regression model, and we identified any evidence of a systematic rise in BP during the year before the event using regression analysis of the most recent premorbid BP vs the log of the time from the BP measurement to the index event, stratified by event type (i.e., lacunar vs nonlacunar). We also compared the long-term maximum BP and CV (≤5 and 15 years before the event) in patients with lacunar vs nonlacunar events using the *t* test. Proportions of patients with premorbid uncontrolled BP using different target levels were also compared in patients with lacunar vs nonlacunar events with the χ^2^ test.

Sensitivity analyses stratified by premorbid use of antihypertensive agent, excluding TIA cases, confined to first-ever-in-a-lifetime incident events and only in patients who had at least 1 BP measurement taken at each time period before the event were also performed.

All analyses were performed with SPSS version 20.

### Standard protocol approvals, registrations, and patient consents

Written informed consent or assent from relatives was obtained in all participants. OXVASC was approved by the local research ethics committee (OREC A: 05/Q1604/70).

#### Data availability

Requests for access to data from OXVASC will be considered by the corresponding author.

## Results

Among 2,555 patients with first-in-the-study-period TIA or ischemic stroke, 331 (12.9%) had events of unknown etiology due to incomplete investigation before death and 90 (3.5%) had multiple etiologies identified after the diagnostic workup and were therefore excluded from the current analysis. Among the remaining 2,134 patients, 2,085 (97.7%; 1,250 stroke, 835 TIA; 309 lacunar events; 493 with age <65 years) had at least 1 premorbid BP recorded (median occasions on which BP measured per patient 16, interquartile range 7–32), with 44,496 premorbid BP measurements in total.

Table 1 summarizes the baseline characteristics of patients with lacunar vs nonlacunar events stratified by age. For patients <65 years of age, known atrial fibrillation and premorbid use of anticoagulants were less common in lacunar vs nonlacunar events, but there were no significant differences in other vascular risk factors or premorbid use of preventive treatment (table 1), including in the prevalence of diagnosed hypertension (46 [48.4%] vs 164 [41.2%], *p* = 0.20; table 1) and premorbid use of antihypertensive agents (32 [33.7%] vs 137 [34.4%], *p* = 0.89; table 1). Among patients ≥65 years of age, those with lacunar events were younger, had less known atrial fibrillation, previous myocardial infarction, and premorbid use of anticoagulants, and more often were current smokers (table 1), but the prevalence of diagnosed hypertension was again similar in lacunar vs nonlacunar events (139 [65.0%] vs 962 [69.8%], *p* = 0.15; table 1), although fewer patients with lacunar events were on premorbid antihypertensive treatment (119 [55.6%] vs 932 [67.6%], *p* = 0.001; table 1).

**Table 1:**
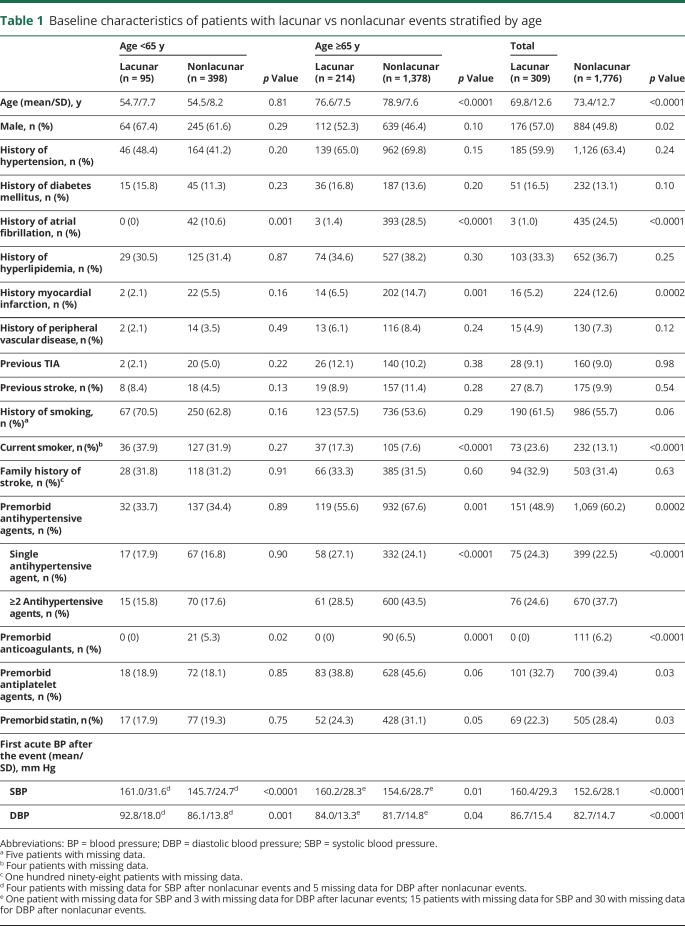
Baseline characteristics of patients with lacunar vs nonlacunar events stratified by age

**Figure 1:**
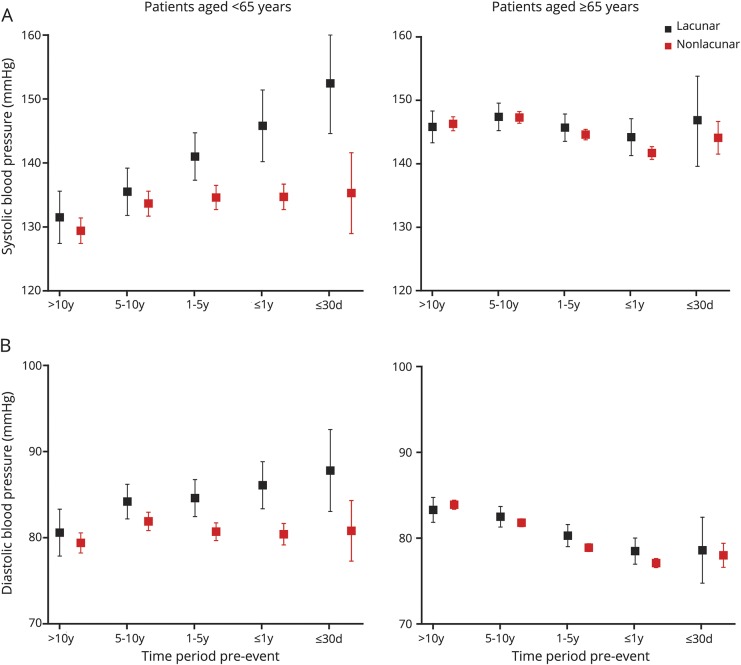
Mean (95% confidence interval) blood pressure taken during different time periods before the index event in patients with acute lacunar vs nonlacunar events stratified by age (A) Systolic blood pressure and (B) diastolic blood pressure.

**Figure 2:**
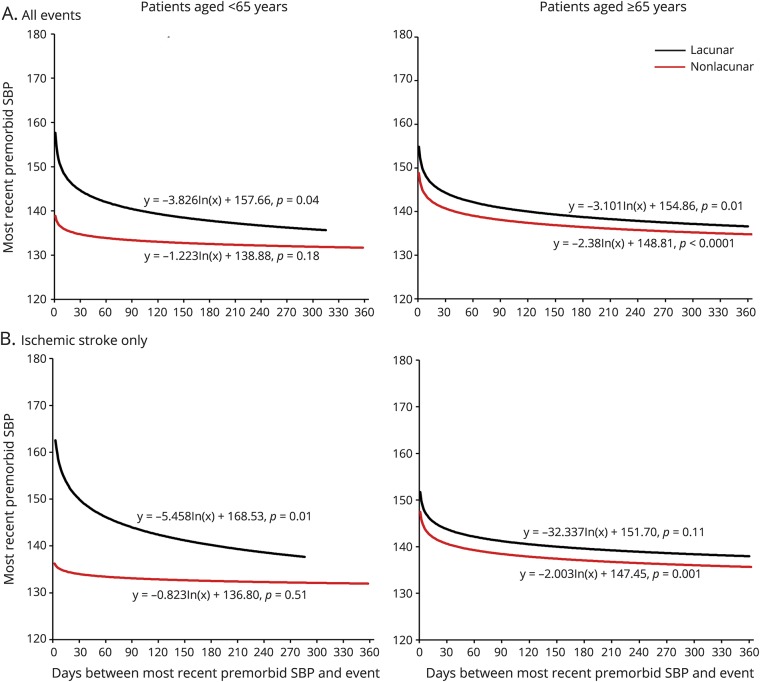
Temporal trends in the most recent SBP measurement during the year before (A) TIA and ischemic stroke or (B) ischemic stroke in lacunar vs nonlacunar events stratified by age Trend lines are derived from a log-linear regression. SBP = systolic blood pressure.

Despite the similar prevalence of diagnosed hypertension in patients with lacunar vs nonlacunar events, for patients <65 years of age, the 15-year mean/SD BP was significantly higher in those with lacunar events (SBP 138.5/17.7 vs 133.3/15.0 mm Hg, *p* = 0.004; DBP 84.1/9.6 vs 80.9/8.4 mm Hg, *p* = 0.001; table 2). Moreover, this difference was explained mainly by a higher mean BP ≤5 years before the event in patients with lacunar events (table 2). When the pre-event time periods were further divided, the difference in BP in patients with lacunar vs nonlacunar events increased (*p*_trend_ = 0.006) with BP measured closer to the index event (figure 1), and the SBP was higher in the weeks and days before (figure 2) and immediately (table 1) after the index event. Sensitivity analyses restricted to patients with at least 1 reading in each time period (table e-1, links.lww.com/WNL/A446), excluding patients with TIA events (table e-2), or confined only to incident events (table e-3) showed consistent results. Trends were also similar in analyses stratified by premorbid use of antihypertensive agents, although it was most prominent in patients on premorbid antihypertensive treatment (15-year mean: SBP 151.8/17.7 vs 141.4/14.9 mm Hg, *p* = 0.001, DBP 91.1/9.6 vs 84.5/8.3 mm Hg, *p* = 0.0001; BP ≤5 years: SBP 153.7/18.8 vs 139.7/16.3 mm Hg, *p* < 0.0001, DBP 90.8/10.3 vs 82.3/9.3 mm Hg, *p* < 0.0001; BP >5 years: SBP 140.5/14.2 vs 142.1/16.4 mm Hg, *p* = 0.65, DBP 87.9/8.0 vs 86.2/8.6 mm Hg, *p* = 0.34; table e-4 and figures e-1 and e-2, links.lww.com/WNL/A445).

**Table 2:**
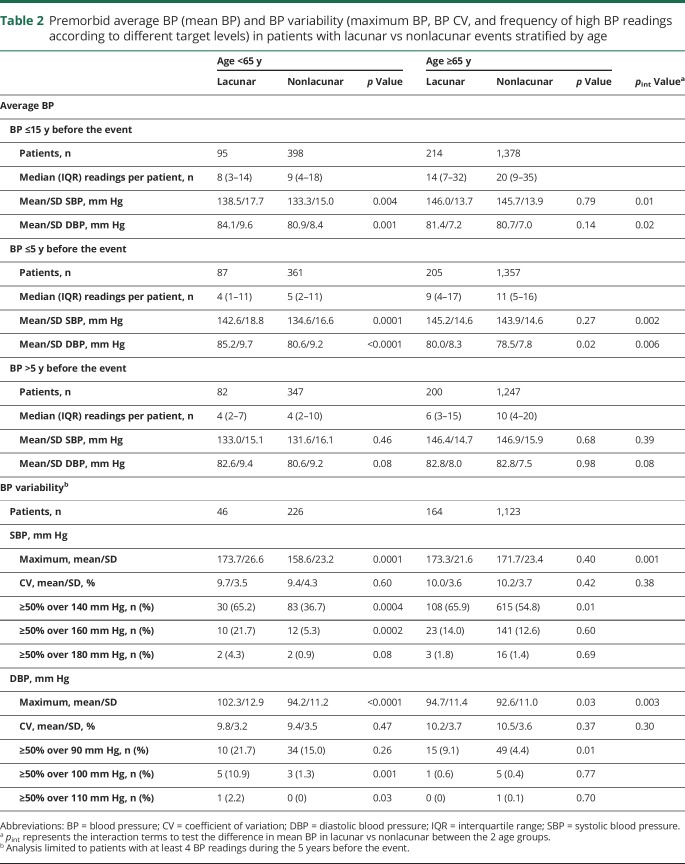
Premorbid average BP (mean BP) and BP variability (maximum BP, BP CV, and frequency of high BP readings according to different target levels) in patients with lacunar vs nonlacunar events stratified by age

In contrast to patients at younger ages, among patients ≥65 years of age, those with lacunar events had similar 15-year mean BP (table 2) or mean BP taken >5 years before the event (table 2) compared to those with nonlacunar events. Although the mean DBP taken ≤5 years before the event was marginally higher for patients with lacunar events (80.8/8.3 vs 78.5/7.8 mm Hg, *p* = 0.02; table 2), the mean SBP taken ≤5 years before the event also did not differ significantly between patients with lacunar and those without nonlacunar events (145.2/14.6 vs 146.9/14.6 mm Hg, *p* = 0.27; table 2), partly as a result of a J-shaped association at BP <110/70 mm Hg (table 3). Sensitivity analyses were again consistent (table e-1–e-4, links.lww.com/WNL/A446). Moreover, when the time periods before the event were further divided, there was no significant difference of BP in lacunar vs nonlacunar events with BP measured closer to the index event (figures 1 and 2; table e-4 and figures e-1 and e-2, links.lww.com/WNL/A445).

**Table 3:**
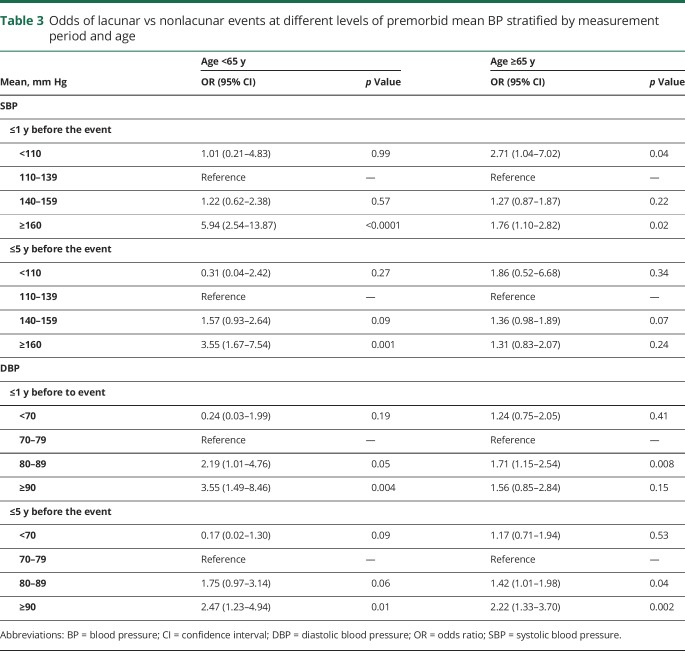
Odds of lacunar vs nonlacunar events at different levels of premorbid mean BP stratified by measurement period and age

In patients <65 years of age, in addition to higher 5-year mean BP for patients with lacunar vs nonlacunar events, the maximum BP in the 5 years before the event was significantly higher in patients with lacunar events (SBP 173.7/26.6 vs 158.6/23.2 mm Hg, *p* = 0.0001; DBP 102.3/12.9 vs 94.2/11.2 mm Hg, *p* < 0.0001; table 2), with no difference in CV (SBP 9.7%/3.5% vs 9.4%/4.3%, *p* = 0.60; DBP 9.8%/3.2% vs 9.4%/3.5%; table 2). Compared to patients with nonlacunar events, those with lacunar events were also more likely to have persistently elevated BP at different target levels (≥50% SBP >160 mm Hg in the last 5 years in lacunar vs nonlacunar; odds ratio 4.95, 95% confidence interval 1.99–12.31, *p* = 0.0002; table 2). However, no similar differences were observed for patients ≥65 years of age (table 2), apart from a marginally higher maximum DBP in patients with lacunar vs nonlacunar events (94.7/11.4 vs 92.6/11.0 mm Hg, *p* = 0.03; table 2). Results were broadly similar for analyses including all 15-year BP measurements (table e-5, links.lww.com/WNL/A446), stratified by premorbid use of antihypertensive treatment (table e-6), excluding TIA events (table e-7), and confined to incident events only (table e-8).

## Discussion

In this population-based cohort of TIA and ischemic stroke with detailed records of premorbid BP, we showed that the associations of BP and acute lacunar events differed by age. Patients with acute lacunar events at younger ages had significantly higher premorbid long-term average BP than those with nonlacunar events, particularly in the 5 years before the index event, with further increases more immediately before the event. This group also had higher maximum premorbid BP and a higher prevalence of uncontrolled BP before the event than patients with nonlacunar etiology. In contrast, the associations of BP and acute lacunar events at older ages were more complex, with possibly different associations of SBP vs DBP and some evidence of a J shape.

Our findings support the hypothesis that recent BP level has a direct role in the etiology of acute lacunar events at younger ages, which is also consistent with previous autopsy studies (mean age ≈65 years) that showed a strong association between hypertension and lacunar infarct^17^ and with the observation that lowering BP is most effective in preventing recurrent stroke in younger patients with recent lacunar infarct.^22^ Of note, we also found the same prevalence of diagnosed hypertension in lacunar vs nonlacunar events, highlighting the fact that the crude prevalence of reported vascular risk factors is not always an adequate measure of risk^23^ and that multiple premorbid BP measurements may be necessary to assess true usual BP.

The findings that the difference in premorbid BP between lacunar and nonlacunar cases was most prominent for BP measurements taken closest to the index event, particularly in patients on antihypertensive treatment, probably reflect a failure to adequately control BP as a result of either treatment failure or noncompliance with treatment.

The diminished associations of BP and lacunar events at older ages are in accordance with a large cohort study that also showed decreased associations of BP and stroke with age.^24^ There are several potential explanations. First, we showed, at older ages, some evidence of a J shape for lacunar vs nonlacunar events at BP <110/70 mm Hg, suggesting that both hypertension and hypotension may play a role in acute lacunar events. However, only 26 patients ≥65 years of age had a 1-year mean SBP <110 mm Hg, so we were not powered to test this hypothesis reliably. Second, our findings support the hypothesis that atheromatous perforator occlusion is more common at older ages, often in association with intracranial stenosis,^18^ whereas acute hypertension induced lipohyalinosis predominates at younger ages. Finally, because hypertension is also a risk factor for atrial fibrillation and large artery atherosclerotic disease, which tend to predominate at older ages, hypertension is likely to be less specifically associated with lacunar stroke at older ages.

We found that compared to nonlacunar events, long-term and recent DBPs were also significantly associated with lacunar events, even at older ages. DBP is sometimes neglected since the shift of clinical focus to SBP in the 1990s.^25^ However, DBP is largely equally informative to SBP in predicting risks of stroke,^2^ particularly at younger ages, and higher DBP is independently associated with lacunes on brain imaging.^11–13,26^ Moreover, there is evidence of a specific association of DBP, independently of SBP, with white matter changes,^27^ microbleeds,^28^ retinal arteriolar narrowing,^29^ and cognitive decline,^30^ indicating that DBP may be an important factor in small vessel disease more generally.^31^

In contrast to mean BP, we did not find significant differences in premorbid BP variability in lacunar vs nonlacunar events at any age. Although BP variability is known to increase the risk of stroke more generally,^32^ it appears not to have a distinct role in acute lacunar events over and above its association with maximum BP, which is consistent with the finding that within-visit BP variability was not associated with recurrent stroke risk in patients with recent lacunar stroke in the Secondary Prevention of Small Subcortical Strokes (SPS3) trial.^33^ We found slightly lower long-term BP variability in lacunar vs nonlacunar events at older ages, possibly reflecting the stronger associations of visit-to-visit BP variability and atrial fibrillation and atherosclerosis,^32^ which are the most common causes for nonlacunar stroke at older ages.

Although we consider the results to be valid, our study has limitations. First, the accuracy of BP can be affected by measurement error. However, inaccuracy of the measurement could not have biased our comparison of lacunar vs nonlacunar events because all measurements were made before the TIA or stroke. Second, a systematic protocol for recording premorbid BP was not possible, and we had to rely on measurements taken in routine clinical practice in primary care. However, this limitation again should not have biased our comparison of lacunar and nonlacunar events. Moreover, because our comparison was stratified by age, the number and timing of premorbid BP readings were also balanced between the groups. Third, the original TOAST classification incorporated etiologic assumptions of hypertension into the criteria for lacunar events.^9^ However, we did not include hypertension and diabetes mellitus in the criteria of our lacunar classification. Finally, we also applied the TOAST classification to classify TIAs. Although not originally developed for TIAs, the usefulness of the TOAST criteria in TIA has been validated.^34^ Moreover, our sensitivity analysis excluding TIAs showed consistent results.

Our findings have several implications. First, we showed that recent premorbid BP control is strongly related to acute lacunar events at younger ages, suggesting that BP plays a direct role in the etiology of small vessel disease at younger ages and highlighting the importance of timely control of BP after diagnosis. Second, DBP should not be neglected, particularly in young and middle-aged individuals, at least from the point of view of small vessel disease prevention. Third, more research is required to understand the more complex associations of BP and lacunar events at older ages. Finally, in studies of other risk factors in the etiology of white matter disease, microbleeds, and associated cognitive decline, our findings highlight the difficulty of excluding confounding by hypertension in the absence of detailed records of prior BP control.

## Glossary

BP: blood pressure
CV: coefficient of variation
DBP: diastolic blood pressure
OXVASC: Oxford Vascular Study
SBP: systolic blood pressure
SPS3: Secondary Prevention of Small Subcortical Strokes
TOAST: Trial of Org 10172 in Acute Stroke Treatment
